# The Future Liver Remnant in Patients Undergoing the Associating Liver Partition with Portal Vein Ligation for Staged Hepatectomy (ALPPS) Maintains the Immunological Components of a Healthy Organ

**DOI:** 10.3389/fmed.2016.00032

**Published:** 2016-08-04

**Authors:** Ram Venkatesh Anantha, Christopher Ryan Shaler, Courtney Erin Meilleur, Jeremy Parfitt, S. M. Mansour Haeryfar, Roberto Hernandez-Alejandro

**Affiliations:** ^1^Department of Surgery, Schulich School of Medicine and Dentistry, Western University, London, ON, Canada; ^2^Department of Microbiology and Immunology, Schulich School of Medicine and Dentistry, Western University, London, ON, Canada; ^3^Department of Pathology, Schulich School of Medicine and Dentistry, Western University, London, ON, Canada; ^4^Division of Clinical Immunology and Allergy, Department of Medicine, Schulich School of Medicine and Dentistry, Western University, London, ON, Canada; ^5^Department of Oncology, Schulich School of Medicine and Dentistry, Western University, London, ON, Canada; ^6^Division of Transplantation, University of Rochester, Rochester, NY, USA

**Keywords:** liver metastases, ALPPS, hepatectomy, liver regeneration, flow cytometry, immunophenotyping

## Abstract

**Background and Aims:**

A short-interval, two-stage approach termed associating liver partition and portal vein ligation for staged hepatectomy (ALPPS) increases the number of patients with extensive malignant disease of the liver and a small future liver remnant (FLR) that can undergo liver resection. While this approach results in accelerated liver hypertrophy of the FLR, it remains unknown whether this phenomenon is restricted to liver parenchymal cells. In the current study, we evaluated whether ALPPS alters the immunological composition of the deportalized lobe (DL) and the FLR.

**Methods:**

In this prospective, single-center study, liver tissue from the DL and the FLR were collected intra-operatively from adult patients undergoing ALPPS for their liver metastases. The extent of hypertrophy of the FLR was determined by volumetric helical computed tomography. Flow cytometry and histological analyses were conducted on liver tissues to compare the frequency of several immune cell subsets, and the architecture of the liver parenchyma between both stages of ALPPS.

**Results:**

A total of 12 patients completed the study. Histologically, we observed a patchy peri-portal infiltration of lymphocytes within the DL, and a significant widening of the liver cords within the FLR. Within the DL, there was a significantly higher proportion of B cells and CD4^+^ T cells as well innate-like lymphocytes, namely mucosa-associated invariant T (MAIT) cells and natural killer T (NKT) cells following ALPPS. In contrast, the frequency of all evaluated immune cell types remained relatively constant in the FLR.

**Conclusion:**

Our results provide the first description of the immunological composition of the human liver following ALPPS. We show that following the ALPPS procedure, while the immune composition of the FLR remains relatively unchanged, there is a moderate increase in several immune cell populations in DL. Overall, our results support the continued utilization of the ALPPS procedure.

## Introduction

Surgical resection remains the only curative treatment option for patients with primary or metastatic malignancies of the liver (1–3). Recently, an innovative procedure termed Associating Liver Partition and Portal vein ligation for Staged hepatectomy (ALPPS) has demonstrated an accelerated hepatocyte regeneration capable of providing good synthetic liver function in patients with a high tumor load and a very small future liver remnant (FLR). However, to date, it remains unknown whether regeneration of the immunological components of the liver also occurs together with the rapidly expanding FLR. Such knowledge is essential to understand the regenerative processes associated with ALPPS and support its utilization.

With extensive intrahepatic tumor involvement present in up to 45% of resectable cases (4, 5), the safety of resection is dependent upon the volume and function of the FLR (6). Patients with inadequate FLR volume and/or function are more likely to experience postoperative liver failure (POLF) and may have an elevated mortality risk (7, 8). Strategies to increase the FLR prior to hepatic resection have typically focused on the manipulation and redirection of portal blood flow by portal vessel occlusion (5, 8, 9). These techniques allow patients with unresectable disease to potentially undergo a major liver resection with a reduced risk of POLF (4, 10–12).

Recently, a short-interval, two-stage approach has been proposed as a viable alternative to portal vein embolism (PVE) in order to rapidly increase the FLR volume in patients with extensive malignant disease in the liver and to remove the macroscopic disease within a short period of time (13–16). Initially, an open right portal vein ligation with *in situ* splitting of the liver parenchyma is performed, while at the same time, the FLR is cleared of metastases as needed. Following a short interval, a second stage is performed to complete the hepatectomy. The ALPPS procedure has been demonstrated to produce accelerated FLR hypertrophy in patients with very small FLR volumes [defined as a liver remnant to body weight ratio (LR/BW) of ≤0.5%] (16–20). Moreover, this technique allows for the removal of the hepatic tumor burden in a shorter timeframe compared to conventional surgical approaches. It also increases the number of patients who can successfully undergo both stages of the procedure. While previous studies have generally reported positive clinical experiences with the ALPPS approach (18–21), demonstrating rapid hepatocyte regeneration and good synthetic liver function, little is known about the implications of the accelerated liver hypertrophy on the resident immune cells present in the liver. Moreover, despite the demonstrated hypertropy following the first stage of ALPPS, it remains unknown whether this phenomenon is restricted to parenchymal cells, or whether resident or recruited immune cell populations will also expand in size in response to this two-stage resection procedure.

Given the importance of the immune system in protecting against infection and cancer development, and in regulating liver repair mechanisms (22–25), understanding how the rapid hypertrophy of the liver affects the immunological content of the liver is of up most importance. More specifically, whether hepatic immune cells of normal liver are present within the FLR and the FLR will remain immunologically competent following the second stage of the ALPPS procedure are essentially unexplored. In the current study, we have enumerated, for the first time to our knowledge, various hepatic immune cell types after the first and second stage of the ALPPS procedure. Our study demonstrates that while moderate immunological changes occur in the DL, the immune constituents of the FLR appear relatively unaltered by the ALPPS procedure.

## Materials and Methods

### Study Design and Setting

This was a prospective, single-center study conducted at the University Hospital (UH), London Health Sciences Centre (LHSC) in London, ON, Canada. Human tissue samples were collected after obtaining written consent from patients undergoing ALPPS at UH. All surgeries were performed by a single hepatobiliary surgeon (RH-A).

Ethics approval for this study was obtained from the Western University Research Ethics Board for Health Sciences Research Involving Human Subjects (Approval number: REB104571), the Lawson Health Research Institute (R-13-490), and the Western University Department of Pathology (TA-830).

#### Patients

All consenting patients, aged 18 and older, with a diagnosis of colorectal liver metastases (CRLM), primary hepatocellular carcinoma, or cholangiocarcinoma, who were admitted to the UH for the ALPPS operation between April 1, 2014 and September 1, 2015, were included in this study (18, 20). Candidates were considered for the ALPPS procedure if they met the following inclusion criteria: technically feasible right trisectionectomy for high liver tumor load; an FLR volume of less than 30%; and the availability of a preoperative computed tomography (CT) scan volumetry.

Patients who were younger than 18 or older than 75 were excluded from this study. Additional exclusion criteria were immunodeficiency, concomitant immunosuppressive therapy, pregnancy, a Do-Not-Resuscitate (DNR) status, an ASA risk score of 3 or higher, an Eastern Cooperative Oncology Group (ECOG) score of 2 or higher, a period of less than 6 weeks between preoperative systemic chemotherapy and surgery, disease progression despite chemotherapy, extrahepatic metastases (e.g., peritoneal, pulmonary, bone marrow, or brain) detected preoperatively or intra-operatively at the time of surgery, tumor involving the left portal or left hepatic veins, preoperative biliary drainage or portal vein obstruction, preoperative signs of systemic or biliary infection (cholangitis), ascites detected *via* ultrasonography, end-stage liver disease, and chronic kidney disease.

#### Surgical Technique

A general laparotomy was performed to rule out extrahepatic disease (18–20). The liver was fully mobilized from the retrohepatic inferior vena cava. An intraoperative ultrasound was performed to assess the technical resectability and for the presence of metastases within the FLR. Once the FLR was cleared of metastases by wedge resections, the right portal vein was identified and ligated while preserving the hepatic arterial flow and biliary drainage of the deportalized liver (DL). The transection lines were placed to the right of the falciform ligament, and complete partition of the liver was performed identifying the right hilar plate. After sufficient hypertrophy of the FLR was obtained (generally within 7–10 days), the right trisectionectomy was completed by a transection of the right hilar plate, as well as the right and middle hepatic veins followed by the removal of the DL.

#### Volumetric Assessment of Hepatic Hypertrophy

All patients underwent volumetric helical CT estimation of their liver volumes before the first and second stages of ALPPS, as per the technique of Farges et al. (10). Measurements were performed for the whole liver, as well as for the right extended liver (including segment 4) and the FLR. The FLR volume was considered to be the volume of the left lateral sector (segments 2 and 3). The estimated rate of the future functional liver remnant volume (%FFLR) was calculated after assuming that the density of the liver was close to 1 by using the following formula: %FFLR = (left liver volume × 100)/(total liver volume − tumor volume) (18–20).

#### Isolation of Non-Parenchymal Hepatic Mononuclear Cells

To obtain non-parenchymal hepatic mononuclear cells (HMNCs), samples of liver tissues (range: 0.1–2 g) were collected from the DL and the FLR during both stages of the ALPPS operation. Freshly isolated tissue was pressed through a 40-μm nylon mesh, and the resulting homogenate was washed in cold PBS, resuspended in a 33.75% Percoll PLUS solution (GE Healthcare Bio-Sciences), and spun at 700 × g for 12 min at room temperature. The supernatant was discarded, and the pelleted cells were resuspended in 2.5% Fetal Calf Serum (FCS) in PBS. Cells were counted using a hemocytometer and resuspended to a concentration of 10 × 10^6^ cells/mL.

#### Flow Cytometry

Freshly isolated, untreated HMNCs (1 × 10^6^ cells/tube) were washed with cold staining buffer (PBS + 2.5% FCS) and stained with diluted fluorescently labeled monoclonal antibodies (mAbs) for the detection of different immune cell types. Cells were stained with various combinations of mAbs to CD3, CD4, CD8, TCR γδ, CD56, TCR Vα24, CD11b, CD14, CD19, CD33, CD68, CD161, and TCR Vα7.2, for the enumeration of various immune cell subsets, including T cell subsets [e.g., CD4^+^, CD8^+^, mucosa-associated invariant T (MAIT) cells, *i*NKT, and γδ T cells], NK cells, B cells, myeloid cells, and myeloid-derived suppressor cells (Table S1 in Supplementary Material). Specific combinations of mAbs were diluted in staining buffer, and the cells were stained at 4°C for 30 min in a volume of 100 μL. Cells were then washed twice with 2 mL staining buffer and incubated with 7-AAD (Table S1 in Supplementary Material) for 5 min. Dead cells were excluded from analysis by gating on 7-AAD^−^ cells. Flow cytometry was performed using a BD FACSCanto II flow cytometer. A minimum of 200,000 events were recorded, and the results were analyzed using FlowJo software (Treestar, Ashland, OR, USA).

### Histological Staining and Assessment

#### Staining

Liver tissue samples from the DL and the FLR at Stages 1 and 2 of ALPPS (1–2 g) were fixed in 10% neutral buffered formalin, embedded in paraffin, sectioned, and stained with hematoxylin and eosin (H&E), or reticulin. For the assessment of the proliferative index, Ki-67 immunohistochemical staining was applied, and an avidin–biotin–peroxidase complex immunohistochemical method was performed for antigen retrieval. The primary anti-Ki-67 antibody (clone MIB-1) was purchased from DAKO, Glostrup, Denmark.

#### Assessment

Sections were examined by a board-certified pathologist specializing in liver diseases (JP), who was also blinded to the study design, procedure stage (stage 1 vs. 2), as well to the liver section from where the tissue was isolated (DL vs. FLR). Ten high-powered visual fields (40×; field diameter 0.55 mm) were examined. Photomicrographs were taken for quantification of the Ki-67 proliferation rate.

### Statistical Analysis

All statistical analyses were performed using GraphPad Prism version 5.03 (GraphPad, La Jolla, CA, USA). Differences between the groups were assessed using the Wilcoxon matched-pairs signed rank test or a Chi-square test for continuous and categorical variables, respectively. In all analyses, a value of *p* ≤ 0.05 was considered statistically significant.

## Results

### Accelerated Liver Hypertrophy in the FLR

**Figure 1:**
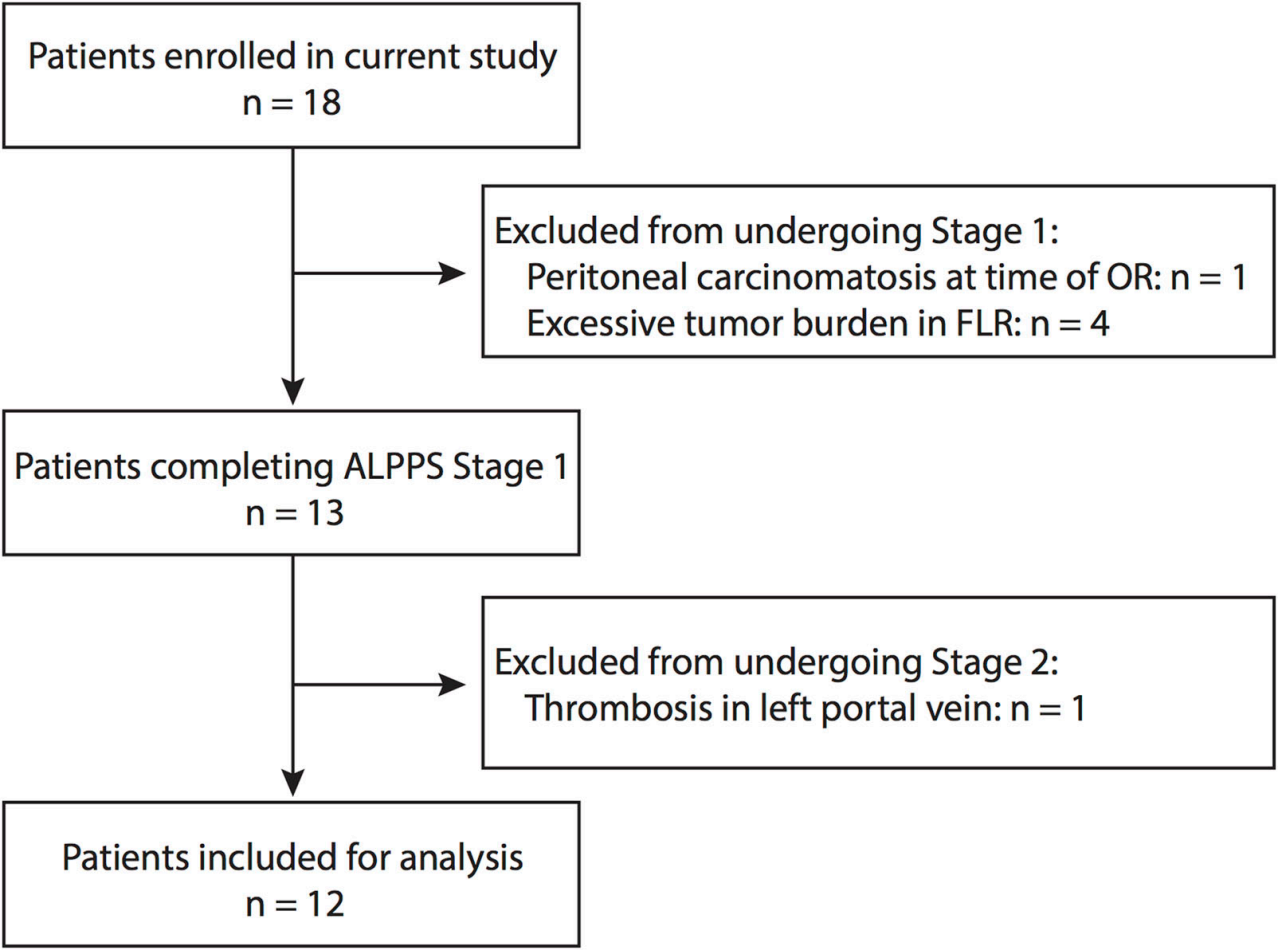
**Patient enrollment for this study**. The provided flow chart outlines at what stages patients were excluded from the study based on the defined criteria.

**Figure 2:**
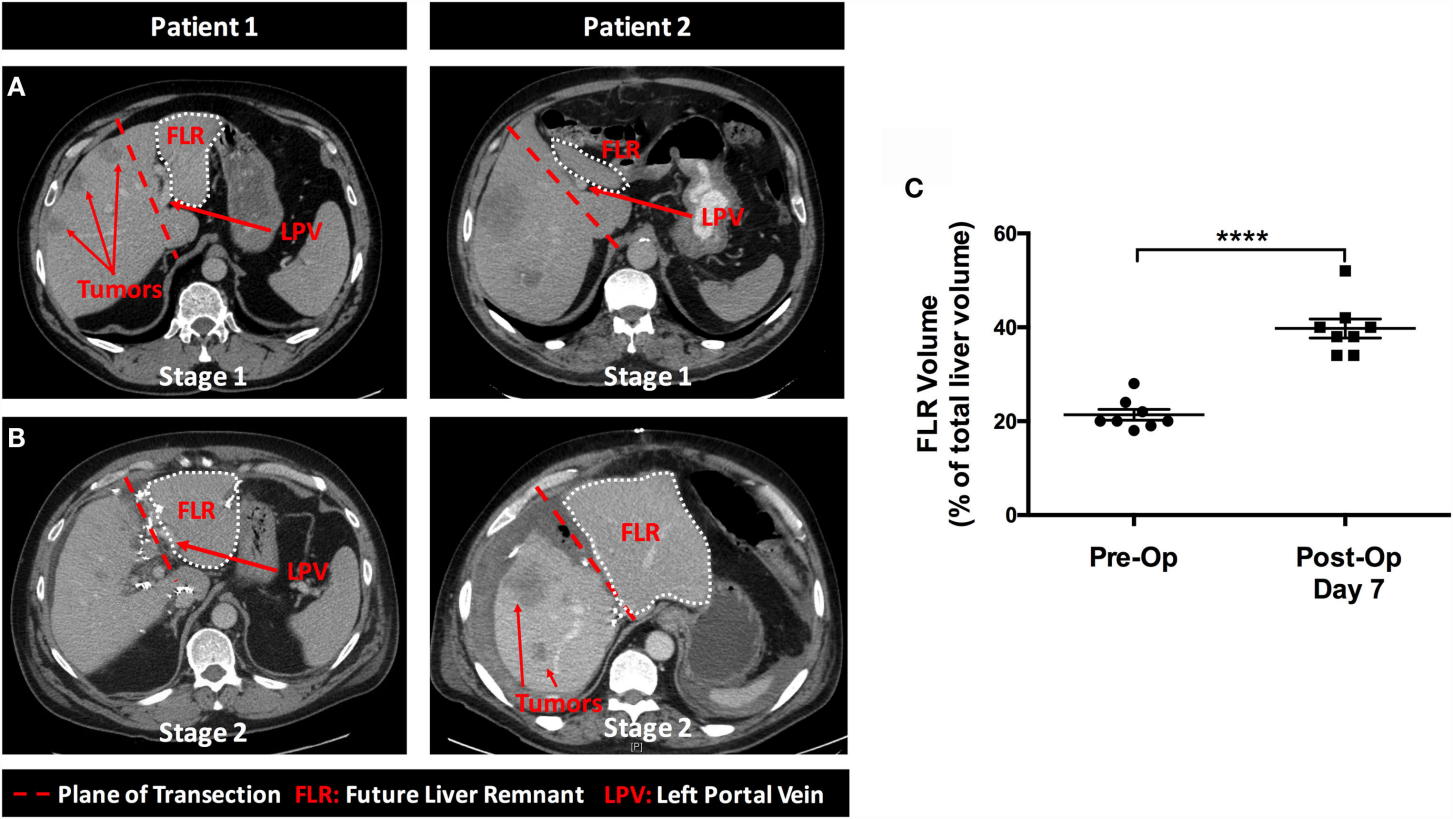
**Rapid hypertrophy of the future liver remnant occurs between stages 1 and 2 of the ALPPS procedure**. In Figure 2, we present a series of representative cross-sectional CT images taken at various stages throughout the ALPPS procedure. **(A)** The burden of malignancy within the liver at the time of diagnosis; **(B)** the magnitude of liver hypertrophy observed following ALPPS Stage 1; and **(C)** a numerical summary of the change in volume for the FLR (expressed as a percentage of total liver volume) pre- and post-ALPPS, as determined by CT volumetry for eight patients.

A total of 18 patients were enrolled in the study. Due to peritoneal carcinomatosis (*n* = 1) or the development of excessive tumor burden in the FLR (*n* = 4), a total of five patients enrolled in the study were precluded from undergoing stage 1. An additional patient, who underwent stage 1, was excluded from the study after developing thrombosis of the left portal vein. Figure 1 shows a flow chart outlining the enrollment of patients in the study. The characteristics of the 12 patients included in our study are reported in Table 1. We routinely performed triphasic CT scans of the abdomen and pelvis at 3 or 7 days after the first stage of the operation on all patients in order to quantify the degree of FLR hypertrophy. In Figures 2A,B, we present a series of representative CT images (annotated to show the major surgical landmarks) for two typical patients at stage 1 (A) and at the time of stage 2 (B). These images are representative of what is typically observed following the first stage of the ALPPS procedure and are summarized numerically in Figure 2C. As depicted in Figure 2C, we observed a significant increase in the FLR volume, relative to the whole liver, between the first and second stage of the ALPPS procedure (from 22 to 39%; *p* < 0.0001), based on CT volumetry. Overall, we demonstrate that there is an accelerated hypertrophy of the FLR following the first stage of the ALPPS procedure.

**Table 1:** **Baseline clinical characteristics and outcomes of 12 patients undergoing ALPPS**.

Variable	Outcome
Clinical characteristics	
Median age, y (±SD)	60 ± 9
Male gender, *n* (%)	7 (58)
Mean body mass index (±SD)	25 ± 3.3
Charlson comorbidity index	6
Oncological characteristics	
Type of primary cancer, *n* (%)	
Colon	4 (33)
Rectum	6 (50)
Other	2 (17)
Synchronous metastases, *n* (%)	6 (50)
Median number of lesions, *n*	2
Number of patients receiving preoperative chemotherapy, *n* (%)	11 (92)
FOLFOX	2 (17)
FOLFIRI	6 (50)
Bevacizumab	6 (50)
Operative characteristics	
Synchronous resection, *n* (%)	7 (58)
Number of patients undergoing simultaneous resection, *n* (%)	3 (25)
Complications, *n* (%)	
All	7 (58)
Severe^a^	3 (25)
Clinical outcomes	
Median volume of FLR pre-ALPPS^c^ (±SEM), %	22 (1.5)
Median volume of FLR post-ALPPS^c^ (±SEM), %	39 (2.7)
Median length of hospital stay, d	18
90-day mortality, *n* (%)	0 (0)
Recurrence^b^ (%)	3 (25)

*^a^Severe complications as defined by the Clavien-Dindo scale (>IIIB)*.

*^b^Includes local, regional, and distal recurrence*.

*^c^Expressed as a percentage of total liver volume*.

### Histological Differences within the DL and the FLR During ALPPS

**Figure 3:**
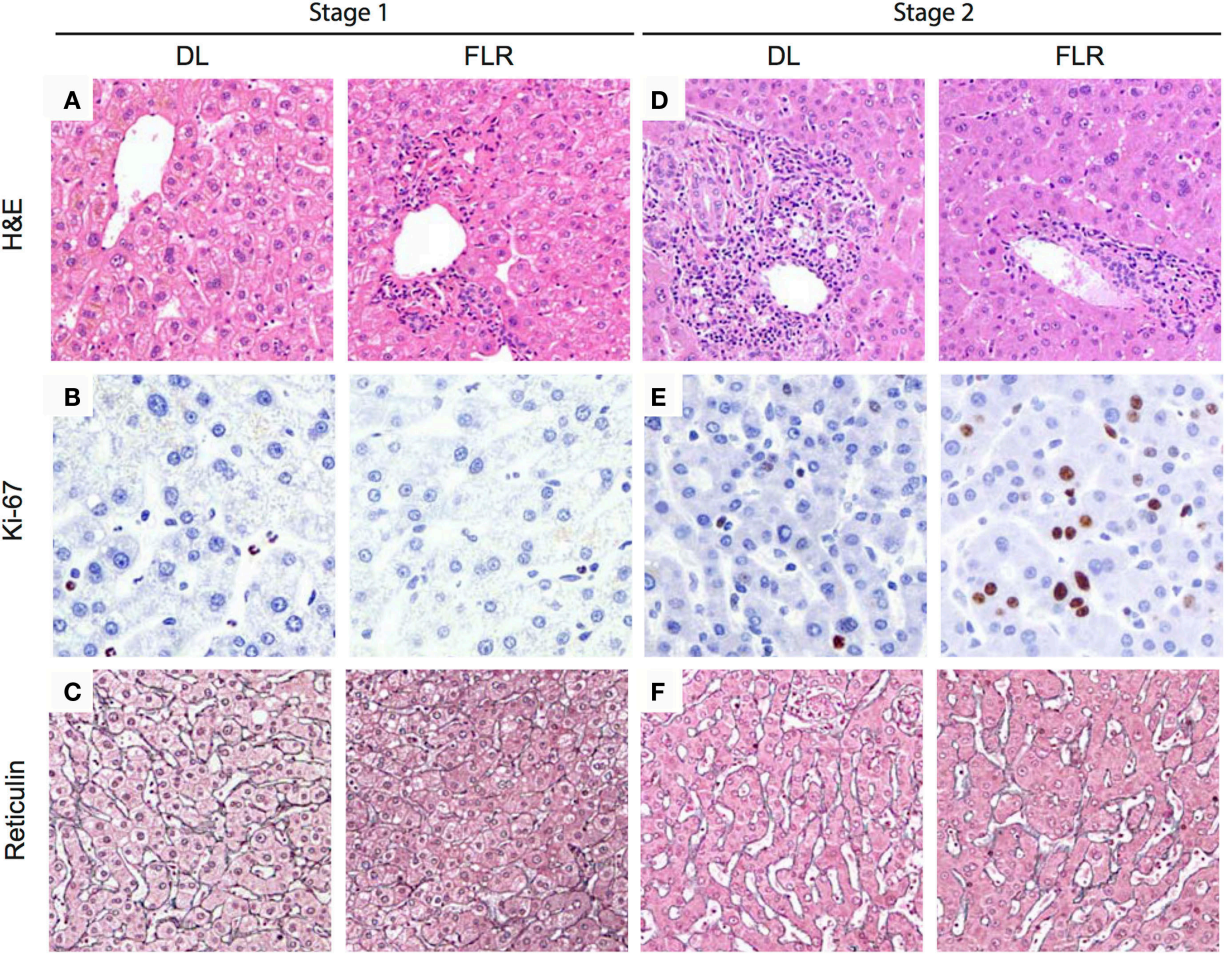
**Histological changes associated with ALPPS at stages 1 and 2 in the DL and FLR**. In Figure 3, we present a series of representative histopathological images for tissues taken at stages 1 and 2. Samples were taken from both the DL and FLR of patients undergoing ALPPS. **(A)** Collected tissues were stained with hematoxylin and eosin (H&E), and the general histopathology of the DL and FLR sections were assessed at stage 1; **(B)** Tissue samples were collected from the DL and FLR at stage 1 and immunostained with Ki-67 to determine basal proliferation in the different liver sections; **(C)** Reticulin staining of samples obtained from the DL and FLR at stage 1; **(D)** Collected tissues were stained with hematoxylin and eosin (H&E), and the general histopathology of the DL and FLR sections were assessed at stage 2; **(E)** Tissue samples were collected from the DL and FLR at stage 2 and immunostained with Ki-67 to determine the overall level proliferation in the different liver sections; and **(F)** Reticulin staining of samples taken from the DL and FLR at stage 2. The presented histology is representative of eight patients undergoing ALPPS, for which histological scoring was performed. Scoring was performed by a board-certified pathologist, and the summary results provided in the text.

We next sought to compare the histopathological differences within the DL and the FLR during ALPPS. Representative histological images are presented in Figure 3. In the first stage, biopsies of the DL and the FLR (prior to the performance of the portal vein ligation and *in situ* liver partition) demonstrated minimal portal inflammation, dilated portal veins, and scattered neutrophils (Figure 3A). The mean Ki-67 proliferation index was less than 1% in both lobes (Figure 3B). Reticulin staining was performed to visualize the reticular fibers comprised of Type III collagen that supports the liver architecture, which demonstrated a normal hepatic plate thickness for both the DL and FLR (Figure 3C).

In stage 2, biopsies of the DL demonstrated patchy portal infiltrate of lymphocytes and eosinophils (Figure 3D). The mean Ki-67 proliferation index within the DL (2.4%) was significantly higher in stage 2 when compared with stage 1 [<1% (*p* < 0.001); Figure 3E]. Reticulin staining of the DL’s architecture demonstrated a slight widening of the liver cords within zone 1, but there were no changes to zones 2 or 3 (Figure 3F). In contrast, the FLR at stage 2 showed portal tracts and central veins that appeared normal histologically, with no peri-portal inflammation or lymphocytosis (Figure 3D). Moreover, the mean Ki-67 proliferation index within the FLR was 3.9%, which was significantly higher than that in Stage 1 (<1%; *p* < 0.01), as well as for the DL at Stage 1 or 2 (*p* < 0.01; Figure 3E). Further, within the liver parenchyma, reticulin staining revealed that the liver cords were significantly wider in the FLR at stage 2 than at stage 1, or when compared to the DL at stage 1 or 2 (Figure 3F). The widening of the liver cords, as identified by reticulin staining, is in line with the observed hypertrophy seen in Figure 2B. Collectively, these results indicate that the hypertrophy associated with ALPPS is largely restricted to the FLR with minimal proliferation in the DL, a finding that is in line with our CT volume measurements (Figure 2C). Overall, after the second stage of the procedure, the architecture of the FLR showed a generally normal appearance.

### Immunological Differences between the DL and the FLR During ALPPS

**Figure 4:**
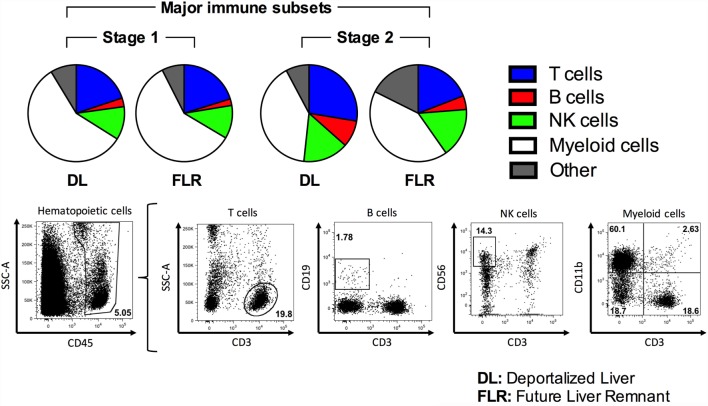
**Comparison of major immune cell populations within the DL and FLR at stages 1 and 2 of ALPPS**. The frequency of various immune subsets was determined by a flow cytometric analysis of non-parenchymal hepatic mononuclear cells isolated from tissue samples collected at stage 1 and 2 from the DL and FLR. A series of pie charts outlining the average proportion of T cells (CD45^+^CD3^+^), B cells (CD45^+^CD3^−^CD19^+^), NK cells (CD3^−^CD56^+^), and myeloid cells (CD45^+^CD11b^+^). All populations were gated off total CD45^+^ hematopoietic cells. Representative dot plots depicting the gating scheme for each of the major immune populations were included.

**Figure 5:**
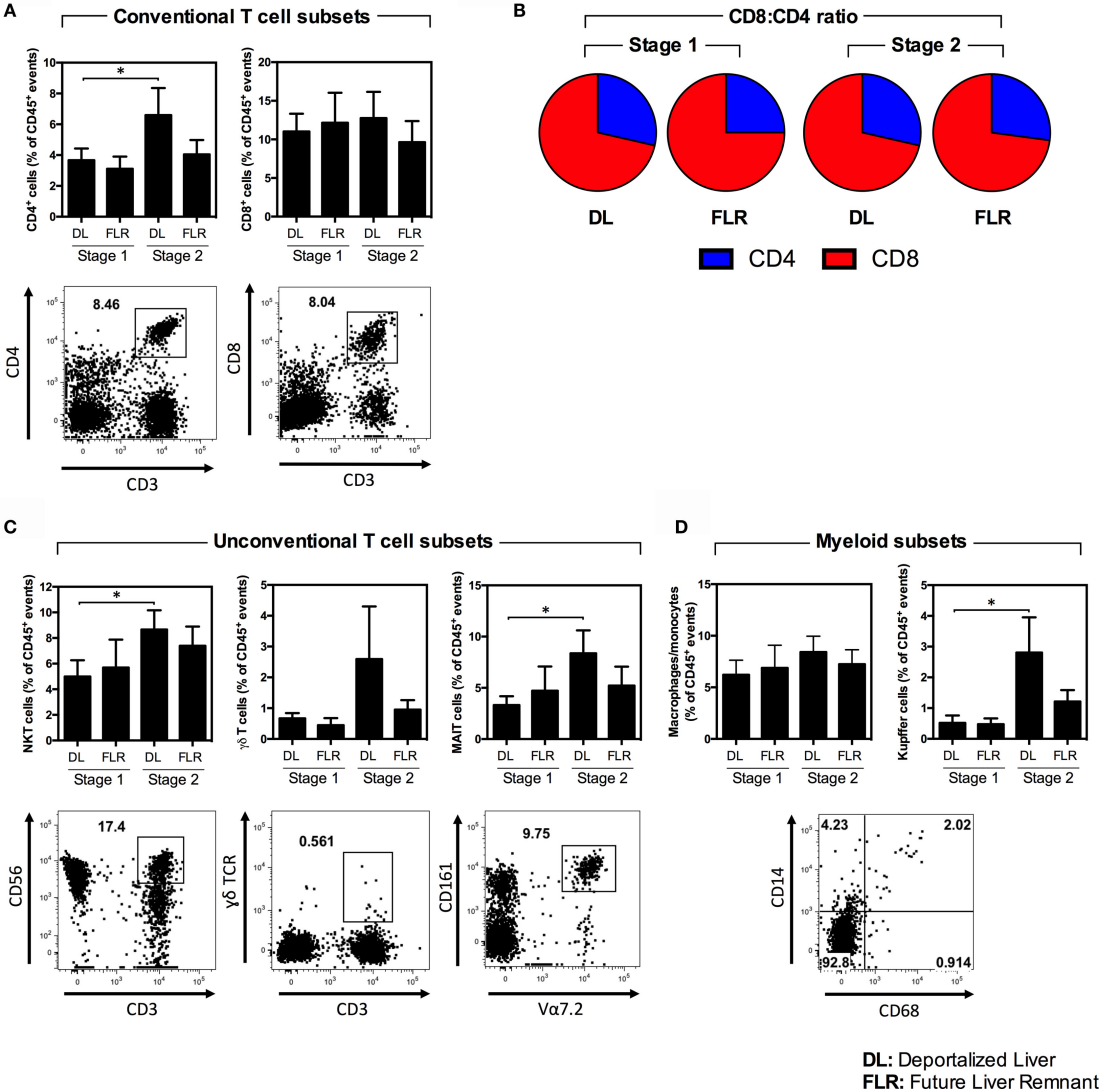
**A comparison of various immune cell subsets within the DL and FLR at stages 1 and 2 of ALPPS**. The frequency of various immune subsets was determined by a flow cytometric analysis of non-parenchymal hepatic mononuclear cells isolated from tissue samples collected at stage 1 and 2 from the DL and FLR. **(A)** The frequency of conventional CD4^+^ (CD45^+^CD3^+^CD4^+^) and CD8^+^ (CD45^+^CD3^+^CD8^+^) T cells; **(B)** The ratio of CD4:CD8 T cells; **(C)** The frequency of the unconventional NKT (CD45^+^CD3^+^CD56^+^), γδ (CD45^+^CD3^+^γδ TCR^+^), and MAIT (CD45^+^CD3^+^Vα7.2^+^CD161^+^) T cells subsets; and **(D)** myeloid cells including Kupffer cells (CD45^+^CD14^+^CD68^+^) and macrophages/monocytes (CD14^+^CD68^−^CD11b^+/−^). Results are the mean ± SEM for a series of six patients undergoing ALPPS. Statistical differences were determined by a Wilcoxon matched-pairs signed rank test *p < 0.05.

We subsequently evaluated the non-parenchymal liver cell populations in the DL and the FLR using flow cytometry. In the first stage, as depicted in Figure 4 and summarized numerically in Table 2, we observed no differences in any of the major immune cell types, including T cells, B cells, NK cells, and myeloid cells when comparing the DL and FLR. Figure 4 depicts the mean frequency of each of the major populations alongside representative dot plots for each of the populations. Moreover, when comparing the DL and FLR during stage 1, we found only minor differences in the frequencies of CD4^+^ and CD8^+^ T cells (Figure 5A), and a similar CD4:CD8 ratio (Figure 5B). Likewise, we observed little differences in the frequency of unconventional T cells subsets including, NKT (CD3^+^CD56^+^), MAIT (CD3^+^Vα7.2^+^CD161^+^), and γδ T cells (CD3^+^γδ TCR^+^) (Figure 5C), as well as the myeloid subsets including Kupffer cells (CD14^+^CD68^+^) and monocytes/macrophages (CD14^+^CD68^−^CD11b^+/−^) when comparing the DL to the FLR (Figure 5D). Representative dot plots for each of the populations are shown below the appropriate bar graph.

**Table 2:** **Mean frequencies of major immune cell populations (expressed as a percentage of all leukocytes) within the deportalized lobe (DL) and the future liver remnant (FLR) during ALPPS**.

Immune markers	Cell type	DL			FLR		
		Stage 1	Stage 2	*p* Value	Stage 1	Stage 2	*p* Value
CD3^+^	T cells	19.9	27.67	0.11	20.22	19.15	0.38
CD19^+^CD3^−^	B cells	2.92	5.35	0.016	1.46	2.42	0.69
CD3^−^CD56^+^	NK cells	11.16	15.21	0.375	11.10	16.57	0.163
CD11b^+^	Myeloid cells	57.4	40.54	0.047	59	42.16	0.22

*Comparison between stages 1 and 2 was made by a Wilcoxon matched-pairs signed rank test*.

In the second stage, the proportions of the majority of the evaluated immune cells did not change in the FLR (Figure 4 and Table 2). In contrast, in the second stage, we observed a general increase in many of the major immune cells in the DL (Figure 4; Figures 5A–D; Table 2). Specifically, we see a significant increase in CD4^+^ T cells, NKT cells, MAIT cells, Kupffer cells, and B cells, as well as a trending increase in γδ T cells and macrophages/monocytes within the DL (Figures 5A–D; Table 2). Overall, we found that while the immunological composition of the FLR remained largely intact following the ALPPS procedure, the DL exhibited moderate increases in several immune cell types. Together, these results suggest that the ALPPS induces regeneration of hepatocytes as well as the resident immune cells in the FLR.

## Discussion

With ALPPS currently being performed in many centers around the world, it is important to understand the implications associated with the accelerated hypertrophy observed in these cases. Here, we demonstrate for the first time that the frequency of many major immune cell types remains largely unchanged in the FLR, whereby the rapid expansion of the FLR occurring between stages 1 and 2 has not resulted in a dilution of liver resident immune cells. Overall, our results provide the first description of the immunological ingredients of the human liver following ALPPS.

The accelerated hypertrophy that occurs after ALPPS has previously been attributed to the diversion of portal blood flow to specific segments, and the abrupt cessation of cross-portal circulation into the DL due to parenchymal transection (14, 16, 26). However, recent animal studies have suggested the potential importance of systemic inflammatory responses in controlling liver hypertrophy following ALPPS (27–29). Schlegel et al. (27) demonstrated that significant liver hypertrophy was induced by parenchymal transection in a mouse model of ALPPS, as opposed to portal vein ligation alone. Moreover, selective injury to distant organs, such as the kidneys and the spleen, also induced significant liver hypertrophy. This suggests that factors secreted into the systemic circulation promoted hypertrophy and regeneration (27, 29). Indeed, Schlegel et al. demonstrated that transferring the plasma collected from mice undergoing an ALPPS procedure into mice undergoing PVE could accelerate the growth of the FLR to levels similar to those observed following ALPPS (27). Together, these observations highlight the important interplay that occurs between the immune system and the parenchymal cells of the liver during the process of liver hypertrophy. Despite this observed cross-talk between the immune system and parenchymal regeneration, the impact of ALPPS on hepatic immune cells has remained largely unexplored.

With the ALPPS procedure able to successfully augment the size of the FLR in a relatively short-time period, it is important to understand whether this rapid hypertrophy alters the distribution and composition of the immune cell populations in the liver. Moreover, the liver is a rich source of innate and adaptive immune cells (24, 25, 30–32), which provide immune surveillance against infection and cancer (33). Furthermore, it has been previously shown that the immune system regulates the repair and regeneration processes of the hepatic parenchyma upon injury (22, 34–36). Therefore, it is important to understand the impact of ALPPS on these populations (23, 37). Specifically, in our current study, we demonstrated that the immune composition of the FLR is largely the same before and after the ALPPS procedure, despite the rapid expansion of the FLR. Importantly, not only did the frequency of various immune cell types not decrease, there was also no substantial increase in the FLR between stages 1 and 2. Specifically, there were no significant increases in any of the evaluated immune cell types in the FLR, a finding that suggests uniform immune cell regeneration. This is important as an increase in the frequency of any one or more immune cell populations may indicate immune activation, potentially inducing inflammation, which may in turn compromise the function of the FLR. However, this suggestion poses a paradox that is the inflammatory process or products may be required for the rapid growth of the FLR, as was suggested by Schlegel and others (27, 34, 38). In contrast to the FLR, in the DL, we observed a generalized but moderate increase in many of the evaluated immune subsets, which may, at least theoretically at this point, contribute to the inflammatory signals thought to drive rapid hypertrophy of the FLR. This is currently a subject of investigation in our laboratory. Moreover, although we only see moderate changes in the frequency of immune cell types in the DL and FLR following ALPPS, we do not currently know what impact this rapid expansion has had on the functionality of these immune populations. Indeed, it would be of interest for future studies to evaluate the differentiation stage of the expanded Kupffer cells or the immunological functionality of the expanded MAIT cells seen in the DL following stage 2.

The underlying reason for applying radical resection is to reduce or eliminate the tumor burden within the liver. Had a decrease been shown for immune cell populations in the FLR, one would have wondered whether this would increase the risk of tumor recurrence or infection due to diminished immune surveillance in this region. Indeed, many immune cell types implicated in tumor surveillance are abundant in the liver.

Comprising up to 10% of the total cell population within the liver (32), lymphocytes, including both T cell subsets such as CD8^+^ Cytotoxic T lymphocytes, NKT cells, and MAIT cells, as well as non-T cell subsets (e.g., NK cells and B cells) are particularly abundant within the liver (39, 40). Importantly, there was no decrease in the frequency of any of the aforementioned populations in the FLR. Interestingly, we also found a mild increase in many of these populations in the DL when comparing their frequencies between stages 1 and 2. While unknown, the expansion of these populations may be due to the reduced blood flow to the region. Indeed, previous studies have demonstrated that many immune cell subsets are remarkably sensitive to hypoxia (41–43). Moreover, the proportions of CD4^+^ T cells, NKT cells, MAIT cells, B cells, and Kupffer cells increased significantly within the DL following ALPPS. Therefore, it remains likely that the activation of these immune cells and the release of soluble inflammatory products in the DL region may contribute to the stimulating factors required for facilitating the rapid hypertrophy of the FLR, as has been previously suggested by others (27). Interestingly, we observed a modest, although non-significant, increase in NK cells in the FLR following the second stage of the procedure. Although many studies have examined the role of NK cells during liver regeneration in animal models, it is still unclear whether they potentiate or suppress hepatic growth (35, 36, 44–47). Graubardt et al. demonstrated that NK cells support hepatocyte proliferation in a mouse model of partial hepatectomy (35). However, Wei et al. observed an inhibition of liver regeneration by NK-cell-induced production of TNF-α in a mouse model of CCl_4_-induced liver injury (44). Additionally, Vujanovic et al. determined that NK cell populations increased significantly during the early phase of liver regeneration following partial hepatectomy in rats (36). However, their activity was functionally suppressed, and this finding potentially represented a regulatory mechanism for which liver regeneration could be enhanced (36). Interestingly, the mechanism by which ALPPS causes rapid hypertrophy of the FLR may be applicable to other models where rapid hypertrophy may be advantageous. Indeed, in one previous report, the size of the transplanted liver graft was successfully augmented through the use of an ALPPS-like procedure in a patient undergoing liver transplantation (48).

Overall, we have demonstrated that following ALPPS, there is rapid hypertrophy accompanied by the presence of all major immune cell types in the regenerated liver. While subtle changes to various immune populations in the FLR were observed, none reached statistical significance. Future studies with a larger sample size are required to validate these changes and to explore the functional capacities of these altered hepatic immune cell types in the FLR between stages 1 and 2 of ALPPS.

## Conclusion

We demonstrate the histopathological and immunological changes that occur within the DL and the FLR following ALPPS in humans for the first time. Our results demonstrate that the liver expansion occurring between stages 1 and 2 of the ALPPS procedure is not only restricted to parenchymal cells but also occurs in resident hepatic immune cells. Together, our results demonstrate that the ALPPS procedure remains a viable alternative to conventional resection techniques by promoting the rapid expansion of the FLR, while allowing the liver to remain immunologically competent. Together, these findings support ALPPS as an innovative surgical option for patients with extensive liver malignancy and a small FLR.

## Author Contributions

RA conceived the study idea, collected the clinical data, participated in the care of the patients, analyzed the data, and contributed to the drafting of the manuscript. CS helped to design and perform the experiments, analyze the data, and drafted the manuscript. CM helped to design and perform the experiments, and analyzed the data. SMMH participated in the study design and helped to draft the manuscript. JP conducted histopathological assessment of tissues. RH-A performed the surgeries for all patients. All authors provided critical revisions of the manuscript for important intellectual content and approved the final version of this manuscript.

## Conflict of Interest Statement

The authors declare that the research was conducted in the absence of any commercial or financial relationships that could be construed as a potential conflict of interest.

## Abbreviations

ALPPS, associating liver partition and portal vein ligation for staged hepatectomy; CRLM, colorectal liver metastases; DL, deportalized lobe; FLR, future liver remnant; POLF, postoperative liver failure; MAIT, mucosa-associated invariant T cell.
