# Supplementary material for: The Future Liver Remnant in Patients Undergoing the Associating Liver Partition with Portal Vein Ligation for Staged Hepatectomy (ALPPS) Maintains the Immunological Components of a Healthy Organ
Source: Front Med (Lausanne). 2016 Aug 4;3:32. doi: 10.3389/fmed.2016.00032 (PMC4972819; doi:10.3389/fmed.2016.00032)
Supplement: Supplementary file 1 [file table_1.docx]

Table S1: List of antibodies used in current study.

| **Antibody/stain/tetramer** | **Fluorochrome** | **Clone** | **Concentration** | **Source** |
| --- | --- | --- | --- | --- |
| 7-Aminoactinomycin D | PerCP | N/A | 20 μL/10^6^ cells | BD Pharmingen |
| CD3 | APC | UCHT1 | 20 μL/10^6^ cells | BD Pharmingen |
| CD3 | FITC | OKT3 | 5 μL/10^6^ cells | eBioscience |
| CD4 | PE | RPA-T4 | 20 μL/10^6^ cells | BD Pharmingen |
| CD8α | FITC | SK1 | 5 μL/10^6^ cells | eBioscience |
| CD11b | FITC | ICRF44 | 5 μL/10^6^ cells | eBioscience |
| CD14 | APC | 61D3 | 5 μL/10^6^ cells | eBioscience |
| CD19 | FITC | HIB19 | 20 μL/10^6^ cells | eBioscience |
| CD33 | PE-Cy7 | WM-53 | 5 μL/10^6^ cells | eBioscience |
| CD45 | Alexa Fluor® 700 | 2D1 | 5 μL/10^6^ cells | eBioscience |
| CD56 | PE-Cy7 | B159 | 5 μL/10^6^ cells | BD Pharmingen |
| CD68 | FITC | eBioY1/82A | 5 μL/10^6^ cells | eBioscience |
| CD161 | APC | HP-3G10 | 5 μL/10^6^ cells | eBioscience |
| hCD1d, unloaded tetramer | APC | N/A | 0.5 μL/10^6^ cells | NIH Tetramer Facility |
| hCD1d, PBS57 tetramer | APC | N/A | 0.5 μL/10^6^ cells | NIH Tetramer Facility |
| TCR-γδ | PE | B1 | 5 μL/10^6^ cells | BD Pharmingen |
| TCR-Vα7.2 | PE | 3C10 | 5 μL/10^6^ cells | BioLegend |
| TCR-Vα24/Jα18 | PE | 6B11 | 5 μL/10^6^ cells | eBioscience |
